# Effectiveness of a family empowerment program on caregiving capacity and adverse mood in caregivers of children with acute leukemia: a quasi-experimental study

**DOI:** 10.1186/s12912-024-01973-2

**Published:** 2024-05-03

**Authors:** Fang Guo, Liangjian Li, Hao Jiang, Jie Yu, Yueqi Wang

**Affiliations:** 1 Department of Nursing, The First Hospital of Jilin University, Changchun, China; 2 China-Japan Union Hospital of Jilin University, Changchun, China; 3 Department of Pediatric Hematology, Children’s Medical Center, The First Hospital of Jilin University, Changchun, China; 4 Department of Neonatology, The First Hospital of Jilin University, Changchun, China

**Keywords:** Family empowerment, Acute leukemia, Caregiver, Caregiving capacity, Illness uncertainty, Anxiety

## Abstract

**Background:**

Family-centered empowerment programs have been widely used in the pediatric field. Therefore, the current study investigated the effectiveness of family empowerment programs on caregiving ability and adverse mood among caregivers of children with acute leukemia.

**Objective:**

To evaluate the effect of a family empowerment program on the caregiving ability and adverse mood of caregivers of children with acute leukemia.

**Methods:**

Sixty-eight children with acute leukemia and their family caregivers admitted to our hospital were selected for the study. The control group received routine care during hospitalization, and the family empowerment program was implemented in the intervention group to compare the changes in caregiving capacity (FCTI), illness uncertainty (PPUS) and anxiety(SAS)of the caregivers of the two groups.

**Results:**

After 8 weeks of intervention, the FCTI score of the intervention group was significantly lower than that of the control group (*P* < 0.001), and the difference between the scores before and after the intervention was statistically significant (*P* < 0.001); the PPUS score of the intervention group was significantly lower than that of the control group (*P* < 0.05), and the difference between the scores before and after the intervention was statistically significant (*P* < 0.001); the SAS score of the intervention group was lower than that of the control group after intervention(*P* < 0.05), and the score difference before and after intervention was statistically significant (*P* < 0.001).

**Conclusion:**

Family empowerment program is beneficial in improving caregiving capacity and reducing disease uncertainty and anxiety among caregivers of children with acute leukemia.

**Trial registration:**

Chinese Clinical Trial Registry ChiCTR2300073476 2023-07-12 Retrospectively registered.

## Introduction

Acute leukemia (AL) is the most common malignancy in childhood, accounting for approximately 28% of the overall malignancy incidence [1]. Among them, acute lymphoblastic leukemia and acute myeloid leukemia are the most common subtypes of childhood and adolescent leukemia [2]. A study showed that about 121,145 children and adolescents in China were diagnosed with cancer between 2018 and 2020, among which leukemia was the most prevalent malignant tumor, with 44.19 per 1 million in boys and 36.72 per 1 million in girls [3].

Once children are diagnosed with AL, they will undergo long-term chemotherapy, during which they usually experience a variety of symptoms due to the disease and chemotherapy drugs, such as gastrointestinal symptoms like nausea, vomiting, loss of appetite, and other symptoms like fatigue and constipation [4, 5]. Due to the young age of the children, their expressive and cognitive abilities are still in the developmental stage, and they do not have the ability to self-manage the disease in the face of complex treatment and severe symptom burden fashion, their treatment and recovery process is mainly dependent on the caregivers, and the role of the caregivers in the care process becomes more prominent [6]. However, some caregivers are at a low level of caregiving ability to undertake caregiving tasks when faced with the shock of a serious illness in a child [7]. Studies [8–10] have found that caregivers lack knowledge and caregiving skills related to the disease and have many needs in the early stages of the diagnosis of the child. Caregivers also suffer from illness uncertainty, anxiety, and depression, which always accompany the caregivers [11, 12]. In addition, caregiving role conflict, impaired socialization, and ineffective coping further contribute to caregiver’s lack of caregiving skills, which directly affects the course of the child’s disease treatment and quality of life [13].

Family Empowerment (FE) is a family-centered, empowerment-based strategic intervention that increases the family’s role in patient health, where health care workers provide medical assistance and partial empowerment to patients and their families, and assist caregivers in acquiring caregiving knowledge, skills, and resources so that they can actively manage behavior change based on health-related needs and effectively assume responsibility for decisions related to promoting and maintaining the health of family members [14, 15].Compared with traditional education models, family empowerment emphasizes a sense of self-management, changing the previous passive form of knowledge education and promoting active participation of patients and caregivers. Deyhoul et al. [16] studied the application of family empowerment to families of stroke patients, leading to significant improvements in patients’ performance of activities of daily living and exercise adherence, and significant improvements in family caregivers’ caring capacity and self-efficacy. In addition, family empowerment has been widely used in the pediatric field to assess its impact on family functioning, disease management skills, and symptoms control in affected children, and significant results have been obtained [17–19].

In summary, the current situation of caregiving among caregivers of children with AL is not optimistic, and there are problems such as low ability to care. Family empowerment can enhance caregivers autonomy and improve caregivers capability to promote the physical and mental health of patients. A study [20] has evaluated its impact on caring capacity and the emotional state of caregivers of children with AL, but its application needs further validation. Therefore, this study aimed to determine the impact of family empowerment on improving caregiving capacity and psychological status of caregivers of children with AL.

## Methods

### Study design

The study was a quasi-experimental research with pre-post test control group. Children with AL and caregivers admitted to the First Hospital of Jilin University were recruited for the study and were divided into a control group and an intervention group according to the time of diagnosis.

### Characteristics of participants

The study included children with AL and caregivers who met the following inclusion criteria: (i) children diagnosed with acute leukemia by clinical diagnostic criteria and within 1 week of starting chemotherapy after diagnosis; (ii) caregiver was a relative who assumed primary care of the child and was conscious and had good reading comprehension and expressive communication skills; (iii) caregivers gave informed consent and voluntarily participated in this study. Exclusion criteria: (i) the child had a combination of other systemic malignant diseases and chronic diseases; (ii) the child was transferred, abandoned treatment or died during the study period; (iii) the carer had a previous history of psychiatric disorders or severe cognitive impairment unable to cooperate with the investigation and study; (iv) the carer participated in other studies or refused to continue to participate in this study.

### Intervention

#### Establishment of family empowerment team

The team consists of a hematologist, a psychological counselor, a nurse practitioner, a pediatric nurse specialist, and a nursing graduate student. The hematologist is responsible for disease diagnosis and treatment, and provides professional guidance for the subject group in the study; the psychological counselor is mainly responsible for the guidance of psychosocial-related knowledge of the subject group; the specialist nurse is responsible for the guidance of nursing knowledge and skills and empowerment education of the subject group; the nursing graduate student is responsible for the initial development of the main contents of family empowerment, and participates in the empowerment education of caregivers in the family empowerment program together with the specialist nurse, and is responsible for recording intervention implementation progress and collecting information. Before the implementation of the project, the subject team members involved in empowerment education were trained and familiar with the content, process and implementation points of the family empowerment program.

### Implementation of the intervention

The intervention group implemented an 8-week family empowerment program, which was scheduled for 1 face-to-face meeting per week after the child was enrolled. The main components of the program were first determined by the researcher based on a literature review [7, 21] and in-depth interviews to understand the caregiver’s care needs to determine the main components of the intervention, and then was reviewed and revised by the team members, mainly covering five topics including disease basics, PICC management, diet management, prevention and management of adverse reactions, and psychological management. The main content was also uploaded to the WeChat applet of the cloud platform “Care for your medical journey” developed by the group for caregivers to learn.

The implementation process is the 5 steps of the Empowering Education Model [15]:

1. Identify the problems - Understand the caregiver’s existing caregiving problems and listen to the caregiver’s experience of caregiving through interactive interviews. Identify caregiving issues for caregivers in the areas of basic disease knowledge, PICC management, recognition and management of adverse reactions, dietary management, and psychological management. Based on the caregiver’s responses, the caregiver’s level of awareness of the illness and the potential for poor caregiving is assessed, and the caregiver is encouraged to think proactively about his or her own problems.
2. Expressing emotions - After establishing the problems, the caregiver is guided to tell the true psychological feelings of the existing stage of the caregiving problem, encouraged to vent out the bad psychological emotions through open-ended questions, listened patiently to the caregiver and gave emotional support to stimulate the caregiver’s sense of self-management.
3. Establishing goals - Based on the caregiver’s existing caregiving problems, such as improper PICC management and specific measures to prevent infection, the caregiver is guided to initiate caregiving goals. The researcher is in a position of appropriate advice and guidance in this process, working with the caregiver to set specific and feasible goals, without forcing goals on the caregiver.
4. Implementation plans - First, the researcher and team members explain the relevant subject matter to the caregiver in response to his or her problems, and ask the caregiver what he or she is prepared to do and give him or her appropriate professional advice, after which both parties participate in developing a corresponding plan in terms of basic knowledge of the disease, PICC management, dietary management, identification and management of adverse reactions, and psychological regulation. Care plans are individualized depending on the receptiveness of the caregiver and his or her situation. In case the caregiver is unclear or has questions about the implementation of the program, the researcher further explain the content or demonstrated the key points of caregiving skills until the caregiver fully mastered them. Caregivers can also view the content in the WeChat applet at any time for easy review.
5. Effectiveness evaluation - After the plan was developed, the researcher followed up with the caregivers daily on the implementation of the plan, answering and recording the caregivers’ questions during the implementation of the plan. When the family empowerment is conducted again, the researcher evaluates the caregiver’s completion of the plan at this stage by asking questions and guiding the caregiver to analyze the implementation of the care plan at the previous stage. The results of the outcome evaluation are also recorded to observe whether the caregiver completed the corresponding plan and achieved the desired goals. During the outcome evaluation process, if the desired goal is met, the next problem intervention is conducted with the caregiver, and if not, the cycle continues with that problem.

### Control group

The control group received routine care and an 8-week verbal teaching session by the charge nurse at the child’s bedside on the same topics as those covered in the Family Empowerment Program, and this content was also made available for caregivers to learn in the form of a WeChat app. Routine care includes: admission education, examination instruction, daily life instruction, medication instruction and discharge instruction.

### Measurement

#### General information questionnaire

The questionnaire included the gender, age, and disease type of the children, age, education level, work status, and monthly family income of the caregivers.

### Family caregiver task inventory (FCTI)

The FCTI was developed by Clark et al. [22] in 1983 and translated into Chinese by Lee et al. [23] of the Hong Kong Polytechnic University in 2011, with a Cronbach’s coefficient of 0.93. Liang Peirong et al. [24] revised it again and applied it to caregivers of children with leukemia, and the Cronbach’s coefficient of the revised scale was 0.887, with good reliability and validity. The scale has 25 entries and 5 dimensions, including adapting to the caregiving role, responding to needs and providing assistance, dealing with personal emotions, assessing family and community resources, and adjusting life to meet caregiving needs. A 3-point Likert scale was used, ranging from 0 to 2, representing “not difficult” to “very difficult”. The total score of the scale was 50, and the higher the score, the lower the caregiver’s ability to care.

### Chinese version of parents’ perception of uncertainty scale (PPUS)

The original scale was developed by Mishel [25] in 1983, with 31 entries. It was revised into a Chinese version by McCarthy et al. [26] in 2014, and the revised scale has a Cronbach’s coefficient of 0.844 and a content validity of 0.928, which has good reliability and validity. The revised scale has 28 entries, including four dimensions of ambiguity, complexity, lack of information, and unpredictability. The scale was scored on a 5-point Likert scale, ranging from 1 to 5 for “strongly disagree” to “strongly agree”, with 9 items being reverse scored, and a total score of 28 to 140, with higher scores indicating greater uncertainty.

### Self-rating anxiety scale (SAS)

The original scale was developed by the scholar Zung [27] in 1971. The scale has 20 items and is rated on a 4-point Likert scale from “no or little time” to “most of the time” on a scale of 1 to 4, respectively. Fifteen items were scored positively, and items 5, 9, 13, 17, and 19 were scored negatively. The scale was based on the standard score results, the total score multiplied by the integer part of 1.25 was the standard score. According to the Chinese normative results, the cut-off value of the standard total score was 50, where < 50 points were no anxiety, ≥ 50 points were mild anxiety, ≥ 60 points were moderate anxiety, and ≥ 70 points were severe anxiety.

### Data collection

The demographic, caregiving capacity, disease uncertainty and anxiety data were collected after the children were definitively enrolled. The caregiving capacity, disease uncertainty and anxiety data were collected again within a week of the end of the intervention.

### Statistical analysis

IBM SPSS Statistics 25.0 software was used for statistical analysis of the data. Descriptive statistics were used to present measurement data as mean ± standard deviation, median and interquartile spacing, and count data as frequency and percentage. The chi-square test and Fisher exact test were used to compare the general data of the two study groups. The Shapiro-Wilk test was used to test the normality of the two groups of researchers before and after the intervention in terms of caregiving ability, uncertainty of illness, self-efficacy and anxiety, and the independent samples t test was used to compare the data that met the normality distribution, and the Mann-Whitney test was used to compare the data that were not normally distributed and the difference was considered statistically significant at *P* < 0.05.

## Results

### Baseline characteristics

A total of 68 children with AL and caregivers were initially included in the study as subjects. During the study, 2 children in the intervention group were transferred to the hospital and 1 child in the control group was transferred to the hospital. The final valid sample consisted of 65 individuals, 32 in the intervention group and 33 in the control group, with a sample efficiency of 94.1%. The comparison of demographic characteristics between the two groups was not statistically significant (*P* > 0.05), indicating a balanced comparability, as shown in Table 1.

**Table 1:** Participants’ demographics

Variables		Intervention group(*n* = 32)	Control group(*n* = 33)	χ^2^	*P*
Child’s gender	Boys Girls	15(46.9) 17(53.1)	19(57.6) 14(42.4)	0.746	0.388
Child’s age (years)	1 ~ 3 4 ~ 7 8 ~ 12 13 ~ 18	6(18.8) 12(37.5) 8(25.0) 6(18.8)	2(6.1) 13(39.4) 15(45.5) 3(9.1)	-	0.175^a^
Singleton	Yes No	15(46.9) 17(53.1)	15(45.5) 18(54.4)	0.013	0.909
Type of disease	Acute lymphoblastic leukemia Acute myeloid leukemia	27(84.4) 5(15.6)	29(87.9) 4(12.1)	-	0.733^a^
Caregiver’s gender	Male Female	4(12.5) 28(87.5)	3(9.1) 30(90.9)	-	0.708^a^
Caregiver’s age (years)	≤ 30 31 ~ 40 41 ~ 50 ≥ 51	4(12.5) 18(56.3) 7(21.9) 3(9.4)	2(6.1) 23(69.7) 5(15.2) 3(9.1)	-	0.687^a^
Education	Junior high school and below High school or secondary school College degree or above	21(65.6) 2(6.3) 9(28.1)	21(63.6) 6(18.2) 6(18.2)	-	0.311^a^
Working condition	Incumbency Not working	8(25.0) 24(75.0)	7(21.2) 26(78.8)	0.131	0.717
Family residence	Countryside Town	15(46.9) 17(53.1)	16(48.5) 17(51.5)	0.017	0.897
Marital status	Married Other	30(93.8) 2(6.2)	30(90.9) 3(9.1)	-	0.515^a^
Income(CNY)	< 1000 1000 ~ 3000 3001 ~ 5000 > 5000	12(37.5) 13(40.6 4(12.5) 3(9.4)	9(27.3) 14(42.4) 6(18.2) 4(12.1)	-	0.799^a^
Medical payment methods	Health care Rural cooperative medical care At your own expense	15(46.9) 15(46.9) 2(6.3)	11(33.3) 21(63.6) 1(3.0)	-	0.429^a^
Religious beliefs	Yes No	2(6.3) 30(93.8)	3(9.1) 30(90.9)	-	1.000^a^

^a^ Fisher’s exact-test

### Main outcomes

The results showed that after 8 weeks of intervention, the FCTI scores were significantly lower in the intervention group than in the control group, with statistically significant differences (*p* < 0.001).The difference in FCTI scores was 8.84 ± 2.17 in the intervention group and 5.70 ± 2.08 in the control group, which was statistically significant. The scores and score differences between the two groups were statistically different (*P* < 0.05) on all dimensions except for the dimensions of assessing family and community resources and adjusting life to meet caregiving needs (*P* > 0.05) (Table 2).

After the intervention, the PPUS scores of the intervention group was significantly lower than that of the control group, and the difference was statistically significant (*P* < 0.05). The difference in PPUS scores was higher in the intervention group than in the control group, and the difference was statistically significantly higher (*P* < 0.001). However, the scores and score differences between the two groups on the complexity and lack of information dimensions were not statistically significant (*p* > 0.05)(Table 3).

The results of the study showed that before the intervention there were 1 caregiver with severe anxiety and 7 caregivers with moderate anxiety in the intervention group, 2 caregivers with severe anxiety and 7 caregivers with moderate anxiety in the control group, and after the intervention there were 0 caregiver with severe anxiety, 0 caregiver with moderate anxiety in the intervention group, and 4 caregivers with anxiety in the control group in the intervention group and the control group. The results of a between-group comparison of the two SAS groups showed that the intervention group had lower SAS scores than the control group, and the difference was statistically significant (*p* < 0.05). The difference in SAS scores was higher in the intervention group than in the control group, with a statistical difference (*p* < 0.001) (Tables 4 and 5).

**Table 2:** Comparison of FCTI Inter- group before and after the intervention

Variables		Before the intervention	After the intervention	Difference
FCTI	Intervention group	23.31 ± 3.70^a^	14.47 ± 2.96 ^a^	8.84 ± 2.17 ^a^
	Control group	24.64 ± 3.86 ^a^	18.94 ± 3.98 ^a^	5.70 ± 2.08 ^a^
	***t***	1.411	5.128	5.961
	***P***	0.163	< 0.001	< 0.001
Adapt to the caring role	Intervention group	5(4,6)^b^	2.5(2,4) ^b^	2(2,3) ^b^
	Control group	5(4,6) ^b^	4(3,5) ^b^	1(1,2) ^b^
	**Z**	0.308	2.506	3.247
	***P***	0.758	0.012	0.001
Respond to contingencies and provide assistance	Intervention group	3(2,3) ^b^	2(1,2) ^b^	1(0.25,2) ^b^
	Control group	3(2,4) ^b^	2(2,3) ^b^	1(0,1) ^b^
	**Z**	1.024	2.924	2.160
	***P***	0.306	0.003	0.031
Deal with personal emotions	Intervention group	6.16 ± 1.46 ^a^	2.97 ± 1.43 ^a^	3.19 ± 1.26 ^a^
	Control group	6.88 ± 1.76 ^a^	4.88 ± 1.80 ^a^	2.00 ± 1.60 ^a^
	***t***	1.796	4.736	3.321
	***P***	0.077	< 0.001	0.001
Assess family and community resources	Intervention group	3(2,4) ^b^	3(2,3.75) ^b^	0.5(0,1) ^b^
	Control group	3(3,4) ^b^	3(2,4) ^b^	0(0,1) ^b^
	**Z**	0.428	0.867	1.019
	***P***	0.669	0.386	0.308
Adjust life to meet care needs	Intervention group	5.91 ± 1.57 ^a^	4.34 ± 1.43 ^a^	1.5(1,2) ^b^
	Control group	6.33 ± 1.47 ^a^	5.06 ± 1.66 ^a^	1(1,2) ^b^
	***t/*** **Z**	1.131	1.866	0.893
	***P***	0.263	0.670	0.372

*Independent samples t test; Mann-Whitney test

^a^$$\bar {X} \pm S$$;^b^*M*(*P*_25_, *P*_75_)

**Table 3:** Comparison of PPUS Inter- group before and after the intervention

Variables		Before the intervention	After the intervention	Difference
PPUS	Intervention group	84.16 ± 7.83 ^a^	70.41 ± 5.68 ^a^	13.75 ± 3.95 ^a^
	Control group	82.64 ± 8.08 ^a^	74.70 ± 8.69 ^a^	7.94 ± 2.76 ^a^
	***t***	0.770	2.364	6.853
	***P***	0.444	0.022	< 0.001
Lack of clarity	Intervention group	36.59 ± 4.83 ^a^	29.38 ± 3.22 ^a^	7(5,9.75) ^b^
	Control group	35.94 ± 5.14 ^a^	32.48 ± 5.67 ^a^	3(2,4.5) ^b^
	***t/*** **Z**	0.529	2.728	4.645
	***P***	0.599	0.009	< 0.001
Complexity	Intervention group	19.47 ± 2.08 ^a^	17.50 ± 2.18 ^a^	2(1.25,2.75) ^b^
	Control group	19.06 ± 2.05 ^a^	17.52 ± 2.09 ^a^	2(1,2) ^b^
	***t/*** **Z**	0.798	0.029	1.881
	***P***	0.428	0.977	0.060
Lack of information	Intervention group	13.03 ± 1.75 ^a^	11.06 ± 1.41 ^a^	2(1,3) ^b^
	Control group	12.64 ± 1.65 ^a^	11.09 ± 1.93 ^a^	2(1,2) ^b^
	***t/*** **Z**	0.935	0.068	1.391
	***P***	0.353	0.946	0.164
Unpredictability	Intervention group	15.06 ± 2.12 ^a^	12.47 ± 1.90 ^a^	2.5(1,4) ^b^
	Control group	15.00 ± 3.07 ^a^	13.61 ± 2.46 ^a^	1(0,2) ^b^
	***t/*** **Z**	0.096	2.081	3.164
	***P***	0.924	0.042	0.002

*Independent samples t test; Mann-Whitney test

^a^$$\bar {X} \pm S$$;^b^*M*(*P*_25_, *P*_75_)

**Table 4:** Comparison of SAS Inter- group before and after the intervention

Variables		Before the intervention	After the intervention	Difference
Anxiety	Intervention group	54.47 ± 7.04 ^a^	45.72 ± 5.90 ^a^	8.75 ± 3.77 ^a^
	Control group	54.55 ± 8.02 ^a^	49.70 ± 7.18 ^a^	4.85 ± 2.21 ^a^
	***t***	0.041	2.436	5.073
	***P***	0.967	0.018	< 0.001

*Independent samples t test

^a^ $$\bar {X} \pm S$$

**Table 5:** Distribution of anxiety levels of study subjects in both groups

Variables	intervention group(*n* = 32)%		control group(*n* = 32)%	
	pre-intervention	post-intervention	pre-intervention	post-intervention
No anxiety	6(18.8)	22(68.8)	9(27.2)	17(51.5)
Mild Anxiety	18(56.2)	10(31.2)	15(45.5)	12(36.3)
Moderate Anxiety	7(21.9)	0(0)	7(21.2)	4(12.1)
Severe Anxiety	1(3.1)	0(0)	2(6.1)	0(0)

## Discussion

The diagnosis of acute leukemia is a huge blow to the entire family of the affected child. Due to the specificity and complexity of the disease, caregivers lack knowledge about the disease early on and have difficulty meeting the care needs of the child [28].At the same time, short-term caregivers suffer from shock and fail to adapt to the treatment needs of their own role, which to a certain extent increases the difficulty of caring for the child, thus inducing complications and reducing the quality of survival of the child [29].The results of this study showed that the difference in FCTI scores was significantly higher in the intervention group than in the control group at the end of the intervention, suggesting that the implementation of family empowerment can improve caregivers’ ability to care, consistent with the results of previous studies [20].With the guidance of family empowerment, caregivers are able to identify their own care problems, gain insight into the nature of the problem through discussion, make them aware of their own shortcomings and the importance of disease management, and stimulate a sense of responsibility for disease management among caregivers. Secondly, family empowerment emphasizes autonomy and empowers caregivers in part to actively engage in caregiving tasks and to motivate their intrinsic motivation. In response to existing care problems, caregivers are guided to set individualized goals, so that they can be reacquainted with disease knowledge and operational skills. This will fully mobilize their subjective sense of initiative and motivate caregivers to deal with problems positively in order to provide better overall care for the children.

Illness uncertainty is a cognitive state in which individuals lack the ability to judge disease-related matters and are unable to organize or categorize disease-related matters [30]. Due to the specificity of the disease, most caregivers lack medical care knowledge about leukemia, and do not understand the treatment, care and disease prognosis of leukemia, coupled with the developed network information about the disease, caregivers cannot form a correct perception of the disease, resulting in a high sense of uncertainty about the disease [31]. The results of this study illustrate the ability to reduce caregiver uncertainty about illness through the implementation of a family empowerment program, consistent with the results of previous studies [32]. The study concluded that the need for information is one of the main reasons for the creation of uncertainty in disease [33]. As an important source of disease information for caregivers, health care professionals should promptly assess caregivers’ perceptions and needs for disease and provide them with targeted information support [34]. In this study, the family of a child with AL was the center of the study, and a harmonious and trusting relationship was established between medical care and caregivers for effective communication. This will identify and assess the caregiver’s needs in a timely manner, address the root cause of the problem, provide valuable information to the caregiver, enable the caregiver to gain in-depth knowledge of the disease and related information, and increase the caregiver’s level of disease awareness, thereby reducing the sense of uncertainty about the disease.

As in previous studies [35, 36], caregivers of children with AL were in a mlid state of anxiety. But caregivers are chronically anxious and their physical and mental health is seriously affected. Sint et al. [37] found that the perceived attitudes of caregivers of children with cancer towards the disease can lead to psychological distress such as anxiety and nervousness in the caregiver. In contrast, this study provides caregivers with disease knowledge and skills through an empowerment process that involves the caregiver in the management of the child’s disease and stimulates a sense of autonomy. At the same time, the study affirmed the progress made by caregivers in disease management, so that caregivers feel benefits in the process to change caregivers’ perceptual perception of disease, thereby reducing caregivers’ anxiety. In addition, medical staff timely assess the psychological state of caregivers, encourage them to express the causes of adverse psychological emotions, and guide caregivers to use psychological relaxation techniques such as meditation and music therapy to reduce anxiety. Therefore, we should always pay attention to the psychological state of caregivers in clinical work, provide them with psychological support and psychological adjustment channels, promote psychological self-regulation of caregivers, reduce anxiety and depression and other emotions to maintain physical and mental health, so as to better care for children and improve their quality of life.

### Limitations

This study has some potential limitations to be aware of. First, this is a single-center experiment with a limited sample size due to factors such as geography, personnel, and time constraints. Therefore, generalizability of results may be limited, and further studies and sample sizes will need to be expanded to confirm this finding. Second, we found that some of the indicators in the study results were not statistically significant, and future studies should extend the study period and recruit more participants to verify the validity of the study. Third, in the evaluation of the intervention effect, the self-assessment questionnaire of the caregiver of the child is used, there are certain subjective factors, and some observation indicators of the child are not involved, and future research should consider the selection and addition of objective evaluation indicators of the child and caregiver to further study the effect of the intervention.

## Conclusion

The results of this study show that family empowerment program is an effective means to improve the care ability of caregivers of children with AL and alleviate adverse emotions. These findings have important implications for nursing practice and provide valuable guidance for the clinical education of caregivers of children with AL.

### Implications for nursing practice and research

The family empowerment intervention improves the caregiving capacity of caregivers of children with AL and reduces caregiver malaise. This intervention promotes a trusting relationship between the nurse and the children’s family, and we believe that this strong bond is critical to improving caregivers’ ability to care and the child’s quality of life. Nurses need to understand the caregiver’s caregiving needs, affirm the caregiver’s decision-making in the face of illness, and provide professional nursing guidance to inspire caregiver confidence and caregiving competence. This intervention could be adopted by pediatric nurses as an essential part of care for children with AL. At the same time, the intervention could be further revised to incorporate mHealth elements to provide guidance to more caregivers of children with AL.

## Data Availability

The datasets used and analyzed during the current study are available from the corresponding author on reasonable request.

## Abbreviations

AL: Acute leukemia
FE: Family Empowerment
FCTI: Family Caregiver Task Inventory
PPUS: Chinese version of Parents’ Perception of Uncertainty Scale
SAS: Self-Rating Anxiety Scale
